# Community-Derived Recommendations for Improving Gender Affirmation of Black and Latine Transgender/Nonbinary Youth

**DOI:** 10.1093/abm/kaae036

**Published:** 2024-07-04

**Authors:** Stanley R Vance, Luz Venegas, Jack Johnson, Anoushka Sinha, Anita V Chaphekar, Jae Sevelius

**Affiliations:** Division of Adolescent and Young Adult Medicine, Department of Pediatrics, University of California, San Francisco, San Francisco, CA, USA; Department of Medicine, Center for AIDS Prevention Studies, University of California, San Francisco, CA, USA; Department of Medicine, Center for AIDS Prevention Studies, University of California, San Francisco, CA, USA; Division of Adolescent and Young Adult Medicine, Department of Pediatrics, University of California, San Francisco, San Francisco, CA, USA; Division of Adolescent and Young Adult Medicine, Department of Pediatrics, University of California, San Francisco, San Francisco, CA, USA; Department of Medicine, Center for AIDS Prevention Studies, University of California, San Francisco, CA, USA

**Keywords:** Transgender youth, Social support, Gender affirmation, Parent–child relations

## Abstract

**Background:**

Gender affirmation is a process by which gender-diverse individuals are supported in their gender identity. Parents are critical in how gender-diverse youth, including Black and Latine transgender/nonbinary youth (BLTY), access various forms of gender affirmation—for example, social and medical transition. Culturally relevant supports are needed to bolster how BLTY and their parents navigate gender affirmation.

**Purpose:**

This study aimed to explore recommendations for aiding BLTY and parents in navigating the youth’s gender journey.

**Methods:**

Semi-structured interviews were conducted with parents of BLTY, BLTY, and BLT young adults (BLTYAs) recruited from clinics, community organizations, and social media. Interviews focused on gender affirmation and recommendations to promote BLTY’s gender affirmation. Primary and secondary analysts coded transcripts using a priori and emergent codes. For this analysis, excerpts pertaining to recommended supports were analyzed to identify themes.

**Results:**

Ten parents of BLTY, 10 BLTY (14–18 years), and 23 BLTYAs (18–30 years) participated. Participants provided recommendations at different socio-ecological levels. On the societal level, participants recommended improvements in media representation of racial and ethnic minority gender-diverse individuals. For organizations, participants recommended more clinicians who shared minoritized identities, clinicians knowledgeable in gender-affirming care, affordability of gender-affirming services, and school-based education regarding gender diversity. On interpersonal/individual levels, they suggested culturally informed peer support among BLTY and parents, including support groups, peer mentors, and camps with individuals who share their minoritized identities.

**Conclusions:**

Participants provided salient insights to supporting gender affirmation of BLTY, which can inform intervention development for BLTY and their families.

## Teaser

Black and Latine transgender/nonbinary youth and their parents made comprehensive recommendations for better supporting the youth’s gender affirmation. These recommendations spanned different domains and can inform future culturally relevant interventions to help families of color support their gender diverse children

## Introduction

Transgender/nonbinary and other gender-diverse youth have gender identities that do not align with societal expectations ascribed to their sex assigned at birth. Unfortunately, these youth experience negative mental health outcomes, including depression, anxiety, mood disorders, and suicidality [1–4] at disproportionate rates compared to their cisgender peers [4–7]. The high burden of mental health symptoms among gender-diverse youth has been observed across socio-ecological domains including medical systems, communities, and schools [2–5, 8, 9]. Moreover, among gender-diverse youth, these symptoms have been found to be associated with experiences of various forms of stigma and marginalization [2, 4, 8, 9]. Notably, systemic transphobia has recently been heightened for these youth due to the increase in enacted local and state laws restricting their rights, including limiting access to gender-affirming medical care [10–12]. This fast-paced proliferation of anti-transgender legislation in the USA has been propagated by movements that vilify gender affirmation.

Black and Latine transgender/nonbinary youth (BLTY) have multiple minoritized identities as they are both racial/ethnic minorities *and* gender diverse. In recent studies, BLTY have been found to have high rates of mental health symptoms, similar to those of White transgender/nonbinary peers [6, 13]. In one school-based study, BLTY and White transgender cohorts had similar odds of past-year depressive symptoms and suicidal thoughts; approximately half of these transgender high school students reported experiencing these symptoms [6]. In a 4-site clinical study, BLTY and White transgender/nonbinary youth had similar rates of depression, but BLTY were *less* likely to report lifetime suicidality [13]. The prevalence of lifetime suicidality for BLTY at 59% was still very high compared to the general adolescent population. For example, in a large U.S.-based study, 12% of adolescents reported lifetime suicidality [14]. Other studies have explored but have not found differences in mental health symptoms comparing BLTY with other race and ethnicity groups among gender-diverse youth [9, 15].

While these studies found that BLTY reported similar rates of mental health symptoms compared to White peers, research that explores unique factors that influence the mental health and well-being of BLTY remains urgent. Firstly, for BLTY, their minoritized identities may be the target of different forms of societal stigma, such as transphobia *and* racism, which intersect and may synergistically impact their mental health and well-being negatively [16–18]. Only recently have studies explored how different forms of stigma in various socio-ecological domains are associated with mental health outcomes for BLTY. For example, school-based survey studies have found that for high school BLTY, experiences of race-, gender-, and sexuality-based harassment are each associated with higher odds of depressive symptoms, suicidal thoughts, and substance use [6, 19].

In addition to understanding the stigma that BLTY experience, research must also identify supportive factors that bolster their well-being. Social-contextual factors that could potentially mitigate mental health risks among BLTY are critical to investigate. For example, a recent study showed that BLTY who reported higher levels of parental acceptance had lower odds of depressive symptoms, similar to their White transgender/nonbinary peers [13]. This study also found that BLTY who did not live socially as their affirmed gender had higher odds of depressive symptoms. These findings are consistent with the Model of Gender Affirmation, which conceptualizes gender affirmation as sociorelational and interactive processes by which gender-diverse individuals receive recognition and support for their gender [20]. The model posits that increased access to various forms of gender affirmation can improve health outcomes among gender-diverse people. For youth, different modes of gender affirmation can be identified across socio-ecological levels [21]. Within the Socio-Ecological Model, individual, interpersonal, organizational (e.g., medical and mental health systems), and societal levels all play critical roles in the development, health, and well-being of young people, including BLTY [21, 22]. Moreover, cultural factors specific to BLTY may impact their access to gender affirmation. Gender norms and expectations within communities and social institutions relevant to BLTY will influence how these youth are supported or not supported in their gender identity.

Unfortunately, studies that focus on gender-diverse youth often do not include racially and ethnically diverse participants and/or fail to measure relevant factors, such as racial and ethnic discrimination, which are important to consider when conducting research on mental health among BLTY [1, 7, 23–29]. Similarly, studies often fail to measure domains that may be uniquely supportive of BLTY, such as racial, ethnic, and cultural pride or community connectedness with individuals who share minoritized identities. To determine relevant support for BLTY and their families, it is imperative to hear directly from them by soliciting their recommendations. By querying them on what would be helpful or not helpful in supporting the BLTY’s gender journey, we can then more directly create culturally informed interventions that target their needs. In this study, we integrate data from two qualitative studies to explore recommendations for interventions that could aid BLTY and their parents in navigating their gender exploration and gender affirmation. The overall goal of this paper is to identify supports to promote the gender affirmation of BLTY in different socio-ecological domains.

## Methods

### Participants and Recruitment

Data reported here are derived from two related qualitative interview studies. For the first study (Young Adult Study), conducted between August 2022 and August 2023, we interviewed BLTYAs. For the second study (Youth–Parent Study), conducted between March 2022 and June 2023, we interviewed BLTY and a related parent separately. Prospective participants were recruited for these studies through (i) community-based organizations (CBOs) and clinics serving gender-diverse clients, (ii) targeted flyering, and (iii) social media campaigns.

For the Young Adult Study, eligible participants were (i) 18–30 years old, (ii) gender diverse, (iii) Black and/or Hispanic/Latine, (iv) able to conduct the interview in English, (v) residing in California, and (vi) not severely cognitively impaired or in active distress. For the Youth–Parent study, youth were eligible if they were (i) 14–18 years old, (ii) gender diverse, (iii) Black and/or Hispanic/Latine, (iv) known to be gender diverse to all parents with whom they resided, (v) able to conduct the interview in English or Spanish, (vi) residing in or receiving care in California, and (vii) not psychiatrically hospitalized in the last month or medically hospitalized in the last 2 weeks. Parents were eligible if they were (i) a parent of the of the eligible youth, (ii) Black or Hispanic/Latine, and (iii) able to participate in English or Spanish.

### Procedures

Prospective participants were screened for eligibility by phone. For the Youth–Parent Study, the parent and youth were screened separately. Informed consent, assent, and/or parental permission were obtained over Zoom; participants provided electronic signatures via DocuSign. For the Young Adult Study, participants provided written informed consent. For the Youth–Parent Study, youth provided written informed assent if they were <18 years old or written consent if they were 18 years old; parents provided written informed consent for their own participation and written permission for youth <18 years old.

Subsequently, participants provided sociodemographic information and were interviewed for approximately 60 min by one of the seven team members. Team members consisted of three people who identify as gender diverse and six people who identify as a person of color; some team members identify as both gender diverse and a person of color. Interviewing team members include individuals with the following professional backgrounds: adolescent medicine physician, adolescent medicine clinical fellow, and professional research associate. The team was under the direct supervision of the senior author who is a clinical psychologist with extensive qualitative methodology experience. The adolescent medicine physician and clinical fellows provide care to gender-diverse youth. They did not interview participants for whom they provide direct clinical care.

For the Youth–Parent Study, the youth and parents were interviewed separately. After the interview completion, each participant was reimbursed a $100 Amazon electronic gift card. Interviews were audio-recorded on the Zoom platform and professionally transcribed in English. Interviews conducted in Spanish were professionally translated. Participants were recruited until saturation was reached with a priori and emergent codes for each study. All files were encrypted and handled according to sensitive material and confidential protocols. The studies were both approved by University of California, San Francisco Institutional Review Board.

### Interview Content

Both studies used semi-structured qualitative interviews to elicit the unique perspectives of Black and Latine gender-diverse individuals and parents of BLTY on a broad range of topics related to gender journeys and various forms of gender affirmation. The interview guides included probes to elicit specific information on these domains: gender exploration (e.g., “Tell me about your first memories of your child being different in terms of their gender.”); gender disclosure and subsequent reaction (e.g., “Tell me about how you went about telling people in your life about your gender identity and how they reacted.”); social transition (e.g., “In what areas in your life have you socially transitioned and what has it been like for you in these areas?”); gender-affirming medical interventions (e.g., “When your child expressed wanting to start medicines for their gender, what did you think of this as a parent?); and multiple intersections of minoritized identities (e.g., “How has your race or culture influenced how people have reacted to your gender?”). Additionally, we asked participants for recommendations for better supporting BLTY and their parents. An example question within this domain was “Imagine that we could create an ideal program to support gender-diverse youth of color and their parents. Tell us how that program would look to you.” Here, we present findings related to these recommendations.

### Analytic Strategy

Transcripts were analyzed with Dedoose [30] using a team-based coding approach with the six team members trained in qualitative analysis under the direct supervision of the senior author. Analysis was conducted using a transcendental phenomenological approach to our interviews, as we aimed to ascertain participants’ reported lived experiences and the meaning behind these experiences. With this approach, we recognize that as researchers, our own personal and cultural lenses will impact our interview data collection. Moreover, we used post-positivism as the interpretive framework as we used preexisting conceptual models in how we analyzed and coded our data. A priori codes were based on the conceptual models that informed the studies, including the Gender Affirmation Model [20]. The Model of Gender Affirmation posits that access to various forms of gender affirmation can improve health outcomes among gender-diverse individuals. Example a priori codes informed by this model pertained to forms of social transition, gender-affirming medical interventions, gender-affirming mental health supports, supports in their gender journey, and challenges to their gender journey.

Another a priori code pertinent to this analysis was “recommended supports” which was applied to interview text that pertained to recommendations for future interventions or activities to support BLTY and their parents. Coding for each study was conducted in parallel with participant recruitment and data collection. Initial coding was conducted by a primary analyst who applied a priori codes. Then, the secondary analyst (Principal Investigator) read each coded interview and provided memos, as needed, to clarify the primary coding. Any differences in code application were resolved with interactive discussions. No major discrepancies occurred, indicating high coder agreement. If either analyst identified new concepts not captured with a priori codes, emergent codes were developed and iteratively applied across interviews using the process described above.

Once all interviews were coded, we conducted searches to identify themes relevant to this analysis and primary study question, “What are meaningful supports and interventions that can aid BLTY and their parents in navigating the youth’s gender journey and gender affirmation?” We searched the “recommended support” code to compile excerpt reports. Focused readings of the excerpt reports resulted in extensive descriptions of themes observed in the data. These descriptive themes were then grouped into higher levels based on their fit into socio-ecological levels. Quotes were selected to represent themes and highlight participants’ recommendations using pseudonyms. We conducted a community check of themes with the Navigating Gender Together Study Community Advisory Board (CAB), comprised of BLTY, parents of BLTY, and service providers in communities of color. The CAB endorsed the resultant themes.

## Results

### Sociodemographic Information

For the Young Adult Study, 23 young adults completed interviews (median age 22; interquartile range (IQR) 4.5 years). Table 1 summarizes other sociodemographic information for young adult participants, who had a wide range of gender identities. For the Youth–Parent Study, 10 youth–parent dyads completed separate interviews for a total of 20 interviews. Table 1 provides a summary of the youth and parent sociodemographic data. BLTY had a median age of 16.5 years (IQR: 1 year). Youth and parents of each dyad reported identical racial and ethnic identities. For both studies, all participants were interviewed in English except for two parents, who were interviewed in Spanish.

**Table 1:** | Study Demographics

Young adult demographics		
		*N* (%)
**Gender identity** ^a^		
Male/transgender Male/transmasculine		8 (35%)
Female/transgender Female/transfeminine		8 (35%)
Nonbinary		7 (30%)
Gender nonconforming		1 (4%)
**Designated sex at birth**		
Female		14 (61%)
Male		9 (39%)
**Race/ethnicity** ^a^		
Latine		14 (61%)
Black		10 (43%)
White		5 (22%)
American Indian/Alaska Native		2 (9%)
South East Asian		3 (13%)
**Gender-affirming medications prior to 18 years old**		7 (30%)
**Currently on gender-affirming medications**		16 (70%)
**Highest attained educational level**		
Less than high school diploma or equivalent		3 (13%)
High school diploma or equivalent		10 (43%)
Associate degree		4 (17%)
Bachelor’s degree		6 (26%)
Youth–parent demographics		
	Youth	Parent
	*N* (%)	*N* (%)
**Gender identity**		
Male/transgender male	7 (70%)	2 (20%)
Female/transgender female	3 (30%)	8 (80%)
**Designated sex at birth**		
Female	7 (70%)	8 (80%)
Male	3 (30%)	2 (20%)
**Race/ethnicity** ^a^		
Latine	6 (60%)	6 (60%)
Black	5 (50%)	5 (50%)
**Currently on gender-affirming medications**	7 (70%)	0 (0%)
**Marital status**		
Married or in domestic partnership	n/a	5 (50%)
Divorced or separated	n/a	2 (20%)
Single/never married	n/a	3 (30%)
**Highest attained educational level**		
Less than high school diploma or equivalent	8 (80%)	0 (0%)
High school diploma or equivalent	2 (20%)	4 (40%)
Some college or vocational/technical school	0 (0%)	1 (10%)
Completed college or vocational/technical school	0 (0%)	5 (50%)

^a^Participants could report more than 1 category; therefore, total percentage is more than 100%.

### Recommendations to Support BLTY and Their Parents by Socio-Ecological Level

**Figure 1.**
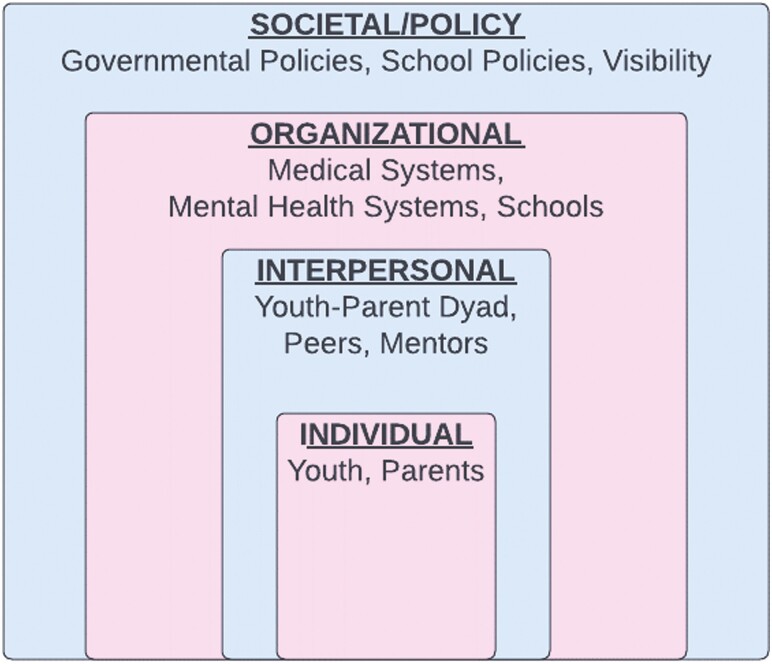
Recommendations to support Black and Latine Transgender/Nonbinary Youth by socio-ecological level

Participants offered a broad range of recommendations for supporting BLTY and their parents that spanned across socio-ecological levels (Figure 1), including (i) societal and policy, (ii) organizational, (iii) interpersonal, and (iv) individual levels.

#### Societal and Policy Level Recommendations

On the societal level, participants made recommendations to bolster positive societal views towards transgender and other gender-diverse individuals. Respondents wanted more societal representation of transgender individuals of color.

I would like to see more representation… I didn’t really know that trans men existed until I started looking it up on YouTube and Instagram and…it was usually White people… it’s not a bad thing, obviously. But it’s so much different when you do see somebody who looks like you. (Cal-Latine, Transgender Male Young Adult)

Participants posited that increased representation would facilitate a youth’s understanding of their gender identity and offer a better sense of what different forms of gender affirmation could look like for someone with their minoritized identities.

There’s not that much information where it comes to…What does it look like for a Black trans person when they start taking testosterone? What is my body going to look like? It’s very hard to find that representation...having more representation for Black and Brown trans people is huge. (Kevin-Black, White, Non-binary, Transmasculine Young Adult)

Additionally, broader representation could have a meaningful impact on the norms of the youth’s family and immediate community. Parents and families of BLTY seeing positive portrayals of transgender/nonbinary youth of color may dispel beliefs that gender diversity is only confined to White communities.

In terms of policy, one parent acknowledged that school and governmental policies that protected her transgender child were an important form of support.

Knowing that there is legislation either at school or in our county that was supporting LGBTQ [individuals] was a support system to us. Because we knew that if we’re feeling resistance or we feel that she’s unsafe…that we knew that there’s legislation and policies to back us up. (Crystal-Black, Female Parent)

This parent recognized the positive role such policies could play as this was her experience. Given the waves of anti-transgender legislation that have markedly increased since our study collection, policies and laws can also be weaponized against transgender youth.

#### Organizational Level Recommendations

Participants provided suggestions to improve the experiences of BLTY and their families engaging in medical and mental health services (Table 2). For some, these recommendations were informed by their own positive experiences; for others, recommendations were based on things they wished they would have experienced.

**Table 2:** | Organizational Level Recommendations to Support Black and Latine Transgender/Nonbinary Youth and Their Families

Recommendation	Description	Exemplar quotations
Increase access to clinical providers who share identities	Access to more clinical providers who share minoritized identities with clients Concordant identities would positively impact clinician’s ability to provide culturally grounded support	“[My psychiatrist] was helpful but she was also like White and cis…She wouldn’t really get it. I would talk to her about my experiences with coming out to my parents and how they kept misgendering me…Counseling for Black, Latinx [youth]…by Black and Latinx therapists. That’s not something that I see.” Eddie-Black, Transgender Male Young Adult “Being able to talk to a queer person who’s a therapist about my queer identity was a game changer. It would be amazing to be able to talk to a Black queer therapist.” Kevin-Black, White, Nonbinary, Transmasculine Young Adult Kevin
Increase access to affirming medical and mental health clinics with knowledgeable providers	Increased access to clinical services with providers knowledgeable in gender diversity and gender-affirming care within primary care clinics and mental health services More affirming support staff and clinical environment Increased gender-affirming services in rural and non-urban areas	“It was like a needle in a haystack trying to find someone that specialized in treating children…You might have to drive seventy miles or a hundred miles out but it’s really worth it to find someone who’s not going to stand in judgment of your child because they don’t understand it.” Debra-Black, Female Parent “Doctor’s offices could be more supportive by whenever you’re going in or signing in by like your pronouns … call the correct name … Or like have an extra gender-neutral bathroom.” Luis-Latine, Male Parent “I’m from a rural area…People who are educated when it comes to gender diverse people and their experiences…would come to not just places like [urban city] and the surrounding areas where there are a bunch of population but smaller areas too.” Carlos-Latine, Transgender Male Young Adult
Improve affordability of gender-affirming medical and mental health services	Access to affordable gender-affirming services, including services that are covered by insurance plans	“I know [that] majority of people who want to transition...do not have the financial ability to do so, I feel, if possible, there should be a way that people who are going to help the child transition and stuff, that there could be some way that they can help them and the family, financially.” Amber-Black, Transgender Female Youth “Definitely having more therapists available that specialize in that, that insurance can cover. Because we don’t have that kind of money.” James-Black, Transgender Male Youth
Increase school education regarding gender diversity	Embedment of education regarding gender diversity and queer history within school curricula Exposure during childhood and adolescent school years may facilitate broader societal normalization of gender diversity and acceptance	“I think queer history needs to be taught because it’s not in schools, at least where I come from. I didn’t grow up here, so I don’t know about here now, but I don’t remember ever hearing about any queer history in my history class when I was growing up. Kids just need to know that these people exist and they’re not outliers to society or people who are on the fringe.” Brenda-Latine, Transgender Female Young Adult “When we have to talk about sex ed, I feel like also having something connected to gender studies is also very important. That way you’re acknowledging the fact that two binaries isn’t the only thing that exists… if you learn it in school, it’ll normalize the fact that it exists.” Len-Black, Nonbinary Young Adult

They recommended more access to clinical providers who share identities (specifically race, ethnicity, and/or gender identity).

Being able to talk to a queer person who’s a therapist about my queer identity was a game changer. It would be amazing to be able to talk to a Black queer therapist. (Kevin-Black, White, Non-binary, Transmasculine Young Adult)

Such providers could provide more culturally informed support and obviate the need for BLTY to educate providers on gender affirmation, transphobia, and/or racism. Increased access to affirming medical and mental health clinics with knowledgeable providers and affirming staff was also endorsed.

It was like a needle in a haystack trying to find someone that specialized in treating children…You might have to drive seventy miles or a hundred miles out but it’s really worth it to find someone who’s not going to stand in judgment of your child because they don’t understand it. (Debra-Black, Female Parent)

“Access” was characterized differently across participants. Some endorsed the availability of more specialized clinics in different geographical locations beyond urban centers. Others wanted clinics to train their staff in both specialty and primary care clinics to be more affirming. Access to gender-affirming services has been identified as critical for helping parents of BLTY engage in their own gender-affirming behaviors [31]. Participants also recommended improved affordability of such services to decrease barriers to access. More school-based education regarding gender diversity was also recommended as a means of improving societal acceptance of gender-diverse individuals.

“When we have to talk about sex ed, I feel like also having something connected to gender studies is also very important. That way you’re acknowledging the fact that two binaries isn’t the only thing that exists… if you learn it in school, it’ll normalize the fact that it exists. (Len-Black, Non-binary Young Adult)

#### Interpersonal and Individual Level Recommendations

Participants provided recommendations for the provision of support on interpersonal and individual levels that would prioritize the BLTY and/or parents. Many participants proposed some form of peer support for the BLTY and parents, such as support from other BLTY, older Black and Latine gender diverse individuals, and parents with their own experiences in navigating the youth’s gender journey and exploration. Ideas for peer support included support groups, peer mentors/coaches, and camps.

For support groups, participants recommended that the primary participants should be BLTY and parents of BLTY with some groups comprised solely of BLTY or parents and other groups with both participating together. Such groups could provide spaces for youth and parents of color to connect on shared cultural experiences, native language, and factors that may impact the youth’s gender journey.

I feel like it would have been really cool to go to youth support groups– but also the ones where your parents can join. I think it would have been cool to have [them in] Spanish… like a Spanish-speaking support group. Just because I feel it would have made it easier for my mom to understand which would have made it easier on me to just be. (Leo-Latine, Transmasculine Young Adult)

Maybe like parents support group…it’s important to respect your kids, but I think it’s also important to talk about the struggles of that transition because it is a transition for them as well from how they refer to their kids basically. (Mateo-Latine, Native American, Non-Binary Young Adult)

My second semester there, somebody started [another group]… I joined it even though the room was a lot smaller. So, it felt more intimate. It felt closer. It was one of those groups that we like kept closed, specifically just for people of color to attend. And I remember feeling I could breathe easier…I actually was able to talk and contribute to conversation. (Cal-Latine, Transgender Male Young Adult)

Some participants recommended a parent support group that included parents who had experience with successfully navigating and supporting their child’s gender journey.

Maybe talking to other parents who have learned to accept their child…Because there’s different cultures that have different expectations for children and for parents. And it would be helpful for them to connect in that way. Maybe a support group. (James-Black, Transgender Male Youth)

One parent described how there were no active support groups or resources in her local rural area. Therefore, they created their own group for support.

Find a tribe for your child, so they can feel supported. We didn’t have that here. We had to create it. We don’t have doctors in town and a Pride center in town. We don’t have any of that in our community. We have gay friends, and we band together; you just try to find support for your child. And I think in doing that, the parent finds support for themselves. (Debra-Black, Female Parent)

Another parent described her experience with online support groups shortly after her child’s gender disclosure. She felt the group did not hold space for parents who were just beginning to understand gender diversity and were not open to empathetic listening. Ultimately, such groups should strive to create safer spaces for parents at all stages of the journey toward acceptance of their transgender/nonbinary child.

[Online groups], some or most of the time may not be helpful if you’re looking for support…It’s really hard for other people to listen to you if you’re not saying what they’re saying or you’re not agreeing with what they’re saying. (Shauna-Black, Female Parent)

Inherent to these recommendations for peer support groups is the notion that there is important knowledge and experiences obtained from a group of similar individuals that is distinct from information provided by clinical service providers. Such support could supplement information provided by gender-affirming clinical providers to aid in the youth’s gender journey.

Another recommended form of peer support involved mentoring and coaching. Peer mentoring is defined as mentoring someone with a similar life experience as oneself [32]. Participants suggested various combinations of peer-to-peer mentoring: (i) Parents early in the process of understanding their child’s gender matched with an experienced parent of BLTY; (ii) BLTY matched with an older Black and Latine gender-diverse individual; (iii) Parents of BLTY matched with an older Black and Latine gender diverse individual. Sharing similar cultural backgrounds and minoritized identities would offer an important source of support to youth and parents as they navigate and cope with various struggles throughout the youth’s gender journey. In some ways, this support is similar to that provided in support groups, but peer mentoring and coaching is 1-on-1, which provides an additional “safe space” to explore struggles.

If parents can have someone to talk to as well as their child have someone to look up to and have a conversation with, I feel like it would be best for everyone—that we all just need some kind of mentor to teach us on … For parents, I feel like they would want another parent who did come up with the same background as them. Or maybe, if they were religious or something, they would want another person who did come from the same religion who did struggle with what they are going through right now but they finally understand it. (Amber-Black, Transgender Female Youth)

Because I feel like when you’re younger a lot of folks experience things like, depression, and anxiety. It can be a lot, like sexuality and gender stuff coming up, too. It would be cool just to see older people who’ve gone through things. (Leo-Latine, Transmasculine Young Adult)

Other participants recommended exclusive or dedicated spaces or events such as summer or get-away camps for BLTY. Such spaces would allow them to take part of a community with individuals with similar minoritized identities and shared experiences. This immersive experience would facilitate peer support.

I feel like a summer camp for…where children can express themselves and start learning about who they are at a young age. And I feel like a summer camp would be perfect. Fun activities like painting or stuff, or some archery, swimming…, There’d be a counselor…a Black, Hispanic counselor, a trans counselor, a gay counselor, and people who are like the kids who can also teach us what they wish they knew when they were at our age. (Taylor-Black, Latine, Transgender Female Youth)

Within these forms of peer support, participants provided informational content that would be specifically useful for BLTY and their families (Table 3), such as education on gender diversity, social transition, and gender-affirming medical care. Other recommended programmatic content included peer advice on effectively advocating for the youth, community building, community empowerment, and promotion of positive future orientation.

**Table 3:** | Peer Support Intervention Content

Information or message delivered	Exemplar quotations
Education regarding gender identity and diversity	“Being able to have people who are either part of the community or like really, really well-versed in gender and sexuality – being able to talk about that with the parents and being able to hold space. Because I want to believe that the world and the people in it are just, more often than not, ill-informed…There is a strong but, ultimately, smaller group of people who are actively trying to be malicious and harmful and want to inflict pain and especially on their own child. So, I think just being able to hold space with people who have the capacity to answer questions like, ‘What does that mean? Like she was born this or, you know, they were born that…’just being able to be, patient and compassionate with that.” Jo-Latine, Filipino Nonbinary Young Adult
Navigating social and medical transition	“I would say once we were in the transition and things like that, just having someone to speak to about it, and also just their advice on how to move forward was really helpful.” Crystal-Black, White, Female Parent
Community-building and empowerment	“It feels very isolating to not see other people who are experiencing the same thing as you…Having more spaces for Black and Brown trans and queer people to be able to find community is really important, too.” Kevin-Black, White, Nonbinary, Transmasculine Young Adult
Navigating various forms of discrimination	“I want to work with more young Black trans kids and queer kids. I would definitely say, ‘Find people that really see you and really hear you and really validate your experiences, because the people that are trying to blow off all those things that you’ve experienced and all the things that you’re noticing that are, you know, anti-Blackness, racism, homophobia.’” Pat-Black, White, NonBinary Young Adult
Promoting positive future orientation	“Having space where folks can get to know one another. But also having like a mentor, like someone who’s older to kind of just even show you the possibility of the future.” Leo-Latine, Transmasculine Young Adult
Advocating for the youth	“You have to be ready to defend your child and advocate for your child and not care what anybody else thinks but your child. Because at the end of the day, if your child doesn’t feel supported by his or her parents, it can be life-ending.” Debra-Black, Female Parent
Bolstering of child–parent relationship	“There would be like a bonding experience, and they get to know their child: what they like and what they don’t like. They can maybe have a different perspective and consider that, ‘Hey, my child’s most happy in this community.’ Or forward people who are like them, they can bond and make friends.” Sal-Latine, Gender-Nonconforming Young Adult

## Discussion

Based on recommendations elicited directly from BLTY, parents of BLTY and BLTYAs, our study’s findings highlight supports that are perceived to be most relevant to the needs of BLTY and their gender affirmation. Recent studies have centered the experiences of BLTY and identified factors such as parental gender affirmation and school-based stressors that impact their psychosocial outcomes [6, 13]. Our current study takes an additional step beyond prior studies’ identification of protective and risk factors for these youth. Youth, young adults, and parents in our study provided ideas to *address* these factors and promote gender affirmation of BLTY in their communities, schools, health care settings, and families.

BLTY must cope with the current challenging and complex sociopolitical climate in which they are experiencing a barrage of stigmatizing and traumatizing messages targeting their identities and rights at multiple levels. As described by one parent participant, local laws and school-based policies that protected her transgender child were critical to her child’s well-being. In her case, these local policies were protective, and she acknowledged the power of laws and policies to impact the youth’s well-being and ability to engage in developmentally critical activities such as school. Presently, a growing number of laws and policies are being enacted in the United States at local and state levels that restrict the rights of transgender youth [10, 11]. Several U.S. states have banned gender-affirming medical care for gender-diverse youth [11]. Such laws impede the rights of parents and youth to make informed medical decisions with their healthcare providers. Furthermore, some states have passed laws prohibiting gender-diverse youth from participating in sports in a manner that is consistent with their gender identity [33, 34]. Admittedly, participants in our study either live or receive care in California, which at the time of this publication, did not have the level of active anti-trans legislation present in other states. Youth and families in states with such marked anti-trans laws and policies may have unique needs to cope with such hostile environments. However, participants in our study and BLTY across the USA are all witnessing their identities being vilified and their ability to live their authentic selves being threatened. In addition to policies directly targeting gender-diverse youth, some U.S. states have increasingly implemented policies restricting education on systemic racism and the history of slavery in the United States [35].

Given these systemic challenges, the identification of meaningful and culturally relevant supports to assist BLTY and their families is especially urgent. An idea that recurred across participant recommendations was the need and desire for BLTY and their families to have exposure to, connections with, and support from other individuals with similar lived experiences and minoritized identities. Having such community with others with shared minoritized identities can be protective and promote resilience building [36]. Such support could potentially impact the mental health of BLTY. Particularly within the family domain, parents receiving encouragement and coaching in engaging in parental gender-affirming behaviors could positively impact the youth’s gender journey and gender affirmation and ultimately their mental health. In a recent qualitative study, BLTY, parents of BLTY, and BLTYAs reported that when parents engaged in gender-affirming behaviors, youth had improvements in mental health, gender dysphoria, confidence in gender identity, and relationships with their parents [31]. These behaviors included name/pronoun use, support for social transition, support of gender-affirming medical interventions, advocacy on the youth’s behalf, and seeking gender-affirming expertise. Although peer support programs have been described for parents of transgender youth, they lack racial/ethnic diversity and empirical evaluation [37, 38]. The participants in our study proposed formats and compositions of such peer supports that they would find helpful. Moreover, they provided informational content that would be specifically useful for BLTY and their families.

These wide-ranging ideas identified in our study for better supporting BLTY can inform the development of programs or augment existing programs in providing culturally informed support for gender-diverse youth of color and their families. For such programs, it will be important for clinics and CBOs to tailor the content and format of such supports based on the local resources, policies, and restrictions. In addition, resources will be needed for the training of staff and peers to provide the content and support required to optimally support BLTY and their families. Finally, it will be critical that these programs be evaluated for their efficacy and impact on BLTY mental health and other outcomes.

At a systems level, participants made suggestions to counter significant barriers to gender-affirming healthcare. One reported barrier particularly relevant to BLTY is the limited number of healthcare providers, who are racial or ethnic minorities or gender diverse. Some participants explained that having clinicians with shared experiences and identities could provide more impactful, culturally informed support. Prior research suggests that having similar provider-client backgrounds and identities are associated with improved client satisfaction with care [39, 40]. This finding reinforces the need for policy makers and health systems to invest in recruiting, training, and retaining clinicians from underrepresented, minoritized groups.

Our study has limitations. Our data were collected from a sample of Black and Latine participants primarily recruited from CBOs and clinics serving gender-diverse youth and families in California. Also, our Young Adult Study was limited to English speakers while our Youth–Parent Study accommodated fluency in English or Spanish for participation. The populations from which we collected data may impact transferability of our findings to individuals of other racial and ethnic backgrounds, who live outside the state of California, and whose primary language is not English or Spanish. Moreover, our recruitment strategy may have introduced selection bias that could yield more representation of resource-connected participants. With our approach, we analyzed interviews of BLTY, parents of BLTY, and BLTYAs instead of separating participants by race and ethnicity. There may have been important differences among racial and ethnic groups in terms of their recommendations that are not explored here.

Despite these limitations, our study presents novel, culturally relevant recommendations provided directly from BLTY, BLTYAs, and parents of BLTY. Our study not only corroborates protective factors shown to be associated with improving youth well-being, but our participants provided prescriptive details for support that can specifically aid them in their gender journey. Embedded in these recommendations is a recurrent theme of desiring to obtain support and gender-affirming care from individuals with shared minoritized identities. Their experiences have not been empirically highlighted in the literature until recently. Our study provides a platform for their voices to be amplified and their ideas for support across domains of their lives.

## References

[1] Olson J , Schrager SM, Belzer M, Simons LK, Clark LF. Baseline physiologic and psychosocial characteristics of transgender youth seeking care for gender dysphoria. J Adolesc Health. 2015; 57(4):374–380. 26208863 10.1016/j.jadohealth.2015.04.027 PMC5033041

[2] Johns MM , Lowry R, Andrzejewski J, et al. Transgender identity and experiences of violence victimization, substance use, suicide risk, and sexual risk behaviors among high school students—19 states and large urban school districts, 2017. Morb Mortal Wkly Rep. 2019; 68(3):67. 10.15585/mmwr.mm6803a3 PMC6348759 30677012

[3] Chen D , Abrams M, Clark L, et al. Psychosocial characteristics of transgender youth seeking gender-affirming medical treatment: baseline findings from the Trans Youth Care Study. J Adolesc Health. 2021; 68(6):1104–1111. 32839079 10.1016/j.jadohealth.2020.07.033 PMC7897328

[4] Wittlin NM , Kuper LE, Olson KR. Mental health of transgender and gender diverse youth. Annu Rev Clin Psychol. 2023; 19:207–232. 36608332 10.1146/annurev-clinpsy-072220-020326 PMC9936952

[5] Guz S , Kattari SK, Atteberry-Ash B, Klemmer CL, Call J, Kattari L. Depression and suicide risk at the cross-section of sexual orientation and gender identity for youth. J Adolesc Health. 2021; 68(2):317–323. 32680801 10.1016/j.jadohealth.2020.06.008

[6] Vance SR, Jr, Boyer CB, Glidden DV, Sevelius J. Mental health and psychosocial risk and protective factors among Black and Latinx transgender youth compared with peers. JAMA Netw Open. 2021; 4(3):e213256. 33769506 10.1001/jamanetworkopen.2021.3256 PMC7998078

[7] Becerra-Culqui TA , Liu Y, Nash R, et al. Mental health of transgender and gender nonconforming youth compared with their peers. Pediatrics. 2018; 141(5):e20173845. 29661941 10.1542/peds.2017-3845 PMC5914494

[8] Perez-Brumer A , Day JK, Russell ST, Hatzenbuehler ML. Prevalence and correlates of suicidal ideation among transgender youth in California: findings from a representative, population-based sample of high school students. J Am Acad Child Adolesc Psychiatry. 2017; 56(9):739–746. 28838578 10.1016/j.jaac.2017.06.010 PMC5695881

[9] Atteberry-Ash B , Kattari SK, Harner V, et al. Differential experiences of mental health among transgender and gender-diverse youth in Colorado. Behav Sci (Basel). 2021; 11(4):48. 33918631 10.3390/bs11040048 PMC8069714

[10] Conron KJ , O’Neill K, Vasquez LA. Prohibiting gender-affirming medical care for youth. Williams Institute at the UCLA School of Law. Available at https://williamsinstitute.law.ucla.edu/publications/bans-trans-youth-health-care/. Accessibility verified November 8, 2023.

[11] American Civil Liberties Union. Mapping attacks on LGBTQ rights in the U.S. state legislatures. Available at https://www.aclu.org/legislative-attacks-on-lgbtq-rights/. Accessibility verified November 8, 2023.

[12] Hughes LD , Kidd KM, Gamarel KE, Operario D, Dowshen N. “These Laws Will Be Devastating”: provider perspectives on legislation banning gender-affirming care for transgender adolescents. J Adolesc Health. 2021; 69(6):976–982. 34627657 10.1016/j.jadohealth.2021.08.020 PMC9131701

[13] Vance SR , Chen D, Garofalo R, et al. Mental health symptoms and gender affirmation among Black and Latine transgender/nonbinary youth prior to initiating gender-affirming hormones. J Adolesc Health. 2023; 73(5):880–886. 37610390 10.1016/j.jadohealth.2023.06.022 PMC10723039

[14] Nock MK , Green JG, Hwang I, et al. Prevalence, correlates, and treatment of lifetime suicidal behavior among adolescents: results from the National Comorbidity Survey Replication Adolescent Supplement. JAMA Psychiatry. 2013; 70(3):300–310. 23303463 10.1001/2013.jamapsychiatry.55 PMC3886236

[15] Turnamian MR , Liu RT. Gender identity and expression in relation to depression and anxiety in racial and ethnic minority youth: evaluations of intersectionality in a population-based study. J Affect Disord. 2023; 339:219–226. 37437727 10.1016/j.jad.2023.07.023 PMC10529835

[16] Turan JM , Elafros MA, Logie CH, et al. Challenges and opportunities in examining and addressing intersectional stigma and health. BMC Med. 2019; 17(1):7. 30764816 10.1186/s12916-018-1246-9 PMC6376691

[17] Sievwright KM , Stangl AL, Nyblade L, et al. An expanded definition of intersectional stigma for public health research and praxis. Am J Public Health. 2022; 112(S4):S356–S361. 35763723 10.2105/AJPH.2022.306718 PMC9241457

[18] Singh AA. Transgender youth of color and resilience: negotiating oppression and finding support. Sex Roles. 2013; 68:690–702.

[19] Vance SR , Boyer CB, Glidden DV, Sevelius J. Comparing substance use and school-based stressors among Black and Latinx transgender youth and peers with shared minoritized identities. J Adolesc Health. 2023; 72(1):44–50. 36224062 10.1016/j.jadohealth.2022.08.029 PMC10204707

[20] Sevelius JM. Gender affirmation: a framework for conceptualizing risk behavior among transgender women of color. Sex Roles. 2013; 68(11-12):675–689. 23729971 10.1007/s11199-012-0216-5 PMC3667985

[21] Szapocznik J , Coatsworth JD. An ecodevelopmental framework for organizing the influences on drug abuse: a developmental model of risk and protection. In: Glantz M, Hartel CR, eds. Drug Abuse: Origins and Interventions. Washington, D.C.: American Psychological Association; 1999: 331–366.

[22] Johns MM , Beltran O, Armstrong HL, Jayne PE, Barrios LC. Protective factors among transgender and gender variant youth: a systematic review by socioecological level. J Prim Prev. 2018; 39(3):263–301. 29700674 10.1007/s10935-018-0508-9 PMC5976555

[23] Allen LR , Watson LB, Egan AM, Moser CN. Well-being and suicidality among transgender youth after gender-affirming hormones. Clin Pract Pediatr Psychol. 2019; 7(3):302–311.

[24] Chen D , Berona J, Chan Y-M, et al. Psychosocial functioning in transgender youth after 2 years of hormones. N Engl J Med. 2023; 388(3):240–250. 36652355 10.1056/NEJMoa2206297 PMC10081536

[25] Turban JL , King D, Carswell JM, Keuroghlian AS. Pubertal suppression for transgender youth and risk of suicidal ideation. Pediatrics. 2020; 145(2):e20191725. 31974216 10.1542/peds.2019-1725 PMC7073269

[26] Chodzen G , Hidalgo MA, Chen D, Garofalo R. Minority stress factors associated with depression and anxiety among transgender and gender-nonconforming youth. J Adolesc Health. 2019; 64(4):467–471. 30241721 10.1016/j.jadohealth.2018.07.006 PMC6528476

[27] Day JK , Fish JN, Perez-Brumer A, Hatzenbuehler ML, Russell ST. Transgender youth substance use disparities: results from a population-based sample. J Adolesc Health. 2017; 61(6):729–735. 28942238 10.1016/j.jadohealth.2017.06.024 PMC6802742

[28] Holt V , Skagerberg E, Dunsford M. Young people with features of gender dysphoria: demographics and associated difficulties. Clin Child Psychol Psychiatry. 2016; 21(1):108–118. 25431051 10.1177/1359104514558431

[29] Kuper LE , Adams N, Mustanski BS. Exploring cross-sectional predictors of suicide ideation, attempt, and risk in a large online sample of transgender and gender nonconforming youth and young adults. LGBT Health. 2018; 5(7):391–400. 30280981 10.1089/lgbt.2017.0259 PMC6425918

[30] Dedoose version 9.0.46. 2021. Cloud application for managing, analyzing, and presenting qualitative and mixed-method research data. Los Angeles, CA: SocioCultural Research Consultants, LLC. Available at https://www.dedoose.com. Accessibility verified November 8, 2023.

[31] Vance SR , Venegas L, Johnson J, et al. Parental Gender Affirmation Model: a culturally informed framework. SSM - Mental Health. 2024; 5:100304. 10.1016/j.ssmmh.2024.100304 PMC11245276 39007080

[32] Pfeiffer PN , Heisler M, Piette JD, Rogers MAM, Valenstein M. Efficacy of peer support interventions for depression: a meta-analysis. Gen Hosp Psychiatry. 2011; 33(1):29–36. 21353125 10.1016/j.genhosppsych.2010.10.002 PMC3052992

[33] Waselewski A , Waselewski M, Waselewski E, Kruger L, Chang T. Perspectives of US youths on participation of transgender individuals in competitive sports: a qualitative study. JAMA Netw Open. 2023; 6(2):e2255107. 36753280 10.1001/jamanetworkopen.2022.55107 PMC9909496

[34] Sin A , Rizzone K, Gonzales G. Sports participation and transgender youths. JAMA Pediatr. 2023; 177(11):1121–1122. 37721743 10.1001/jamapediatrics.2023.3266

[35] Natanson H. Few legal challenges to laws limiting lessons on race, gender. The Washington Post. 2023. https://www.washingtonpost.com/education/2023/03/17/legal-challenges-gender-critical-race-theory/. Accessibility verified November 8, 2023.

[36] Romijnders KA , Wilkerson JM, Crutzen R, Kok G, Bauldry J, Lawler SM; Montrose Center. Strengthening social ties to increase confidence and self-esteem among sexual and gender minority youth. Health Promot Pract. 2017; 18(3):341–347. 28420269 10.1177/1524839917690335

[37] Thornburgh C , Kidd KM, Burnett JD, Sequeira GM. Community-informed peer support for parents of gender-diverse youth. Pediatrics. 2020; 146(4):e20200571. 32973119 10.1542/peds.2020-0571 PMC7546091

[38] Hillier A , Torg E. Parent participation in a support group for families with transgender and gender-nonconforming children: “Being in the company of others who do not question the reality of our experience.”. Transgend Health. 2019; 4(1):168–175. 31406916 10.1089/trgh.2018.0018 PMC6689185

[39] Takeshita J , Wang S, Loren AW, et al. Association of racial/ethnic and gender concordance between patients and physicians with patient experience ratings. JAMA Netw Open. 2020; 3(11):e2024583. 33165609 10.1001/jamanetworkopen.2020.24583 PMC7653497

[40] Ku L , Vichare A. The association of racial and ethnic concordance in primary care with patient satisfaction and experience of care. J Gen Intern Med. 2023; 38(3):727–732. 35688996 10.1007/s11606-022-07695-y PMC9186269

